# Data integration and evolutionary analysis of long non-coding RNAs in 25 flowering plants

**DOI:** 10.1186/s12864-021-08047-6

**Published:** 2021-10-14

**Authors:** Shiye Sang, Wen Chen, Di Zhang, Xuan Zhang, Wenjing Yang, Changning Liu

**Affiliations:** 1 CAS Key Laboratory of Tropical Plant Resources and Sustainable Use, Xishuangbanna Tropical Botanical Garden, Chinese Academy of Sciences, Menglun, Mengla, 666303 Yunnan China; 2 College of Life Sciences, University of Chinese Academy of Sciences, Beijing, 100049 China; 3 Center of Economic Botany, Core Botanical Gardens, Chinese Academy of Sciences, Menglun, Mengla, 666303 Yunnan China; 4 The Innovative Academy of Seed Design, Chinese Academy of Sciences, Menglun, Mengla, 666303 Yunnan China

**Keywords:** lncRNAs, Flowering plants, Evolutionary analysis, Conservation, *Arabidopsis thaliana*, Co-expression network

## Abstract

**Background:**

Long non-coding RNAs (lncRNAs) play vital roles in many important biological processes in plants. Currently, a large fraction of plant lncRNA studies center at lncRNA identification and functional analysis. Only a few plant lncRNA studies focus on understanding their evolutionary history, which is crucial for an in-depth understanding of lncRNAs. Therefore, the integration of large volumes of plant lncRNA data is required to deeply investigate the evolution of lncRNAs.

**Results:**

We present a large-scale evolutionary analysis of lncRNAs in 25 flowering plants. In total, we identified 199,796 high-confidence lncRNAs through data integration analysis, and grouped them into 5497 lncRNA orthologous families. Then, we divided the lncRNAs into groups based on the degree of sequence conservation, and quantified the various characteristics of 756 conserved *Arabidopsis thaliana* lncRNAs. We found that compared with non-conserved lncRNAs, conserved lncRNAs might have more exons, longer sequence length, higher expression levels, and lower tissue specificities. Functional annotation based on the *A. thaliana* coding-lncRNA gene co-expression network suggested potential functions of conserved lncRNAs including autophagy, locomotion, and cell cycle. Enrichment analysis revealed that the functions of conserved lncRNAs were closely related to the growth and development of the tissues in which they were specifically expressed.

**Conclusions:**

Comprehensive integration of large-scale lncRNA data and construction of a phylogenetic tree with orthologous lncRNA families from 25 flowering plants was used to provide an oversight of the evolutionary history of plant lncRNAs including origin, conservation, and orthologous relationships. Further analysis revealed a differential characteristic profile for conserved lncRNAs in *A. thaliana* when compared with non-conserved lncRNAs. We also examined tissue specific expression and the potential functional roles of conserved lncRNAs. The results presented here will further our understanding of plant lncRNA evolution, and provide the basis for further in-depth studies of their functions.

**Supplementary Information:**

The online version contains supplementary material available at 10.1186/s12864-021-08047-6.

## Background

Long non-coding RNAs (lncRNAs) are a class of non-coding RNAs longer than 200 nt with high tissue specificities. They can be divided into intergenic, intronic, sense and antisense lncRNA transcripts based on their relative position to coding genes [1]. In recent years, a variety of methods based on machine learning algorithms have greatly improved the performance of lncRNA identification and functional annotation, and made lncRNA function study in diverse species receive extensive attention, such as the significant roles of lncRNAs in tumorigenesis and cancer progression, and in plant development and stress responses [2–5]. The sequence homology and conservation of lncRNAs provide insights into their functions. Therefore, there is a need to understand the evolutionary dynamics of lncRNAs.

In animals, great breakthroughs have been made in lncRNA evolutionary analysis, which is of great benefit to the understanding of the functions of animal lncRNAs and the evolution of regulatory networks in which they are involved. Washietl et al. found that mammalian long intergenic non-coding RNAs (lincRNAs) show strong conservation of tissue specificities and higher primary sequence conservation in promoters and exons than in evolutionarily young lincRNAs [6]. An analysis of evolutionary age and lncRNA families in tetrapods showed that ancient lncRNAs, which were generally actively regulated, might play a major role in embryonic development, and conserved lncRNAs probably function in fundamental processes including spermatogenesis and synaptic transmission [7]. Interestingly, Hezroni et al. reported that the conserved functions of lincRNAs required only short patches of specific sequences and could withstand major changes in gene structure [8].

In plants, no large-scale comprehensive evolutionary analyses of lncRNAs have been performed in multiple species, but some attempts have been made. A comparison between *A. thaliana* lincRNAs and genomic sequences of the other six plant species showed that only 2% of lincRNAs displayed evolutionary conservation [9]. Additionally, Li et al. revealed that only 25% of the lncRNAs identified in maize (*Zea mays*) could find homologous in sorghum (*Sorghum bicolor*) [10]. A recent study reported that 575 orthologous lncRNA pairs were identified between *A. thaliana* and *Arabidopsis lyrata*, while few orthologous lncRNA pairs were identified in rice (*Oryza sativa*) and it’s four related species [11].

A large number of lncRNAs have been discovered in various plants. LncRNA data resources are rapidly growing, and have made it possible to use bioinformatics methods and tools to collect and integrate lncRNA data to study its evolution and function. Here, we conducted a large-scale evolutionary analysis of lncRNAs from 25 flowering plants. Through data integration analysis of four public lncRNA databases, we identified 199,796 high confidence lncRNAs, and classified them into orthologous families. We grouped these lncRNAs based on their sequence conservation, and compared the sequence, structure and expression differences between conserved and non-conserved lncRNAs to produce a comprehensive profile of conserved plant lncRNAs. This work allows us to better understand the evolution of plant lncRNAs, and provides valuable clues for further in-depth studies of plant lncRNA functions.

## Results

### Comprehensive collection and integration of lncRNAs in 25 flowering plants

To analyze plant lncRNAs as comprehensively as possible, we used an analysis pipeline with three main parts: 1) lncRNA data collection and integration; 2) lncRNA sequence conservation analysis; and 3) lncRNA functional annotation. For the first part, we collected lncRNAs from four comprehensive and reliable public plant lncRNA databases, including CANTATAdb2.0, GreeNC, RefSeq, and NONCODE. LncRNA sequences were then filtered based on sequence length (longer than 200 nt), coding potential (calculated by CPC2) and genome location (identified by Gffcompare). Based on the amount of data, and the quality of the genomes and genome annotations, we further selected 25 species for subsequent analysis. The obvious overlap between different data sources required us to integrate the data from different databases to obtain a non-redundant lncRNA dataset. This was the key step in the first part of our pipeline. The lncRNA sequences were mapped to specified genome versions of the plants to determine their exact genomic locations, and redundant lncRNAs were removed if their locations were highly overlapping (Additional file 1). As a result, we obtained a final high confidence lncRNA dataset containing 199,796 lncRNA transcripts (Table 1).

**Table 1:** lncRNA data integration results

Species	GREENC	CANTATADB	RefSeq	NONCODE	Number of lncRNAs collected	Number of unique lncRNAs
*Amborella trichopoda*	5698	5511	4750	0	15,959	7074
*Arabidopsis lyrata*	4363	7593	5311	0	17,267	9363
*Arabidopsis thaliana*	3008	4373	4083	3763	15,227	5539
*Brachypodium distachyon*	5584	4945	8779	0	19,308	6783
*Brassica napus*	0	12,010	18,114	0	30,124	16,597
*Brassica rapa*	0	8501	10,102	0	18,603	10,797
*Citrus sinensis*	2562	0	6581	0	9143	3430
*Cucumis sativus*	1929	7348	1639	0	10,916	5466
*Fragaria vesca*	3503	0	5007	0	8510	3889
*Glycine max*	6689	3096	10,980	0	20,765	8817
*Gossypium raimondii*	4216	0	12,765	0	16,981	6422
*Malus domestica*	4126	10,924	11,608	0	26,658	9228
*Manihot esculenta*	3468	9504	4874	0	17,846	7660
*Medicago truncatula*	9676	3590	3874	0	17,140	10,904
*Musa acuminata*	4071	3001	5428	0	12,500	5121
*Oryza brachyantha*	0	6004	2720	0	8724	4926
*Oryza sativa*	5237	2788	10,090	0	18,115	7211
*Populus trichocarpa*	5569	4322	0	0	9891	8334
*Prunus persica*	3301	2902	7274	0	13,477	4183
*Solanum lycopersicum*	3440	4716	2203	0	10,359	6807
*Solanum tuberosum*	6680	5790	7727	0	20,197	7797
*Sorghum bicolor*	5305	2600	4821	0	12,726	6326
*Theobroma cacao*	4268	5256	9026	0	18,550	7459
*Vitis vinifera*	2526	4542	5340	0	12,408	6151
*Zea mays*	18,110	10,761	11,543	0	40,414	23,512

In the 25 species examined, lncRNAs of seven species came from two databases, lncRNAs of 17 species came from three databases, and lncRNAs of *A. thaliana* came from four databases. Moreover, there was a 6.85-fold variation in the numbers of lncRNAs in different plants, from 3430 in *Citrus sinensis* to 23,512 in *Zea mays*. Species with more than 10,000 lncRNAs also include *Brassica napus*, *Brassica rapa*, and *Medicago truncatula*, while other species each have lncRNAs in the thousands.

### Plant lncRNA sequence conservation

**Fig. 1:**
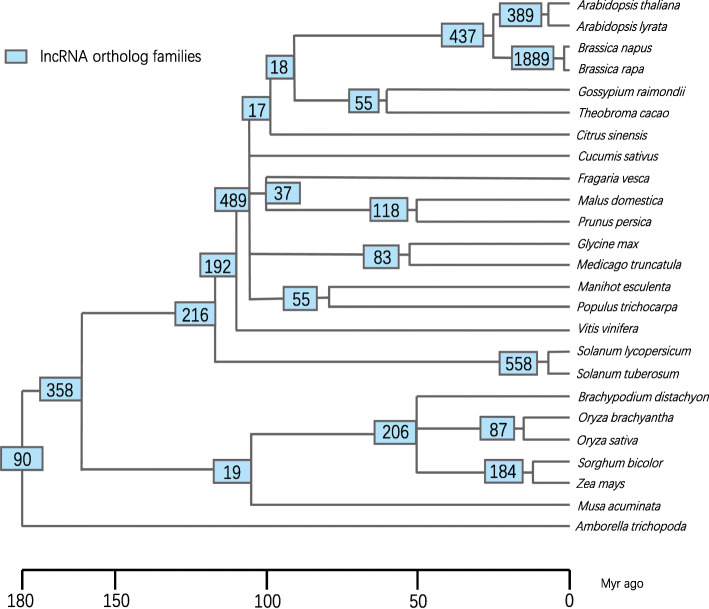
The phylogenetic tree of lncRNA orthologous families. The numbers in the blue box at the internal branches and roots represent the number of lncRNA orthologous families

Putative lncRNA orthologous families were identified in plants through multi-species comparison. BLASTN was used to perform pairwise alignment of lncRNA sequences between species [12]. lncRNA orthologous pairs were identified through reciprocal best hits, and they were connected using the single-linkage clustering method to construct lncRNA families. To further explore the evolutionary conservation of plant lncRNA, all lncRNA families were located on a phylogenetic tree made by TimeTree according to which species does lncRNAs in them belong to (Fig. 1) [13].

The phylogenetic tree revealed that the evolution of the lncRNAs in these 25 species spans 180 Myr. We identified 5497 lncRNA families with a total of 13,564 conserved lncRNAs. Among these lncRNA families, 1953 (35.5%) families had a total of 6269 (46.22%) conserved lncRNAs that originated more than 50Myr ago, 1401 (25.49%) families had a total of 5171 (38.12%) conserved lncRNAs that originated more than 100Myr ago, 448 (8.1%) families had a total of 2381 (17.55%) conserved lncRNAs that originated more than 150 Myr ago, and 90 (1.64%) families had a total of 692 (5.10%) conserved lncRNAs that originated more than 180 Myr ago.

More detailed statistics revealed that only 6.79% (13,564 lncRNAs) of the collected lncRNAs (199,796 lncRNAs) were conserved across the plant kingdom. In *A. thaliana*, the percentage of conserved lncRNAs was 15.56%. The highest proportion of conserved lncRNAs was observed in *Brassica rapa* (21.01%) and the lowest in *Amborella trichopoda* (1.30%) (Additional file 2). These findings suggest that plant lncRNAs have a fast evolutionary rate, resulting in poor sequence conservation. Further investigation of the proportion of conserved lncRNAs at each branch point revealed that the proportion of conserved lncRNAs among related species (within the same genus) tends to be larger than the proportion of conserved lncRNAs between distant species (between the genera or more distant relatives). The number of orthologous families between *Brassica napus* and *Brassica rapa* is 1889, and their percentage of conserved lncRNAs is the highest (13.79%). This indicates that most lncRNAs were traced to more closely related ancestors, and makes us guess that lncRNAs between closely related species were more likely to be conserved.

### Differences between Arabidopsis lncRNAs with conserved and non-conserved sequences

**Fig. 2:**
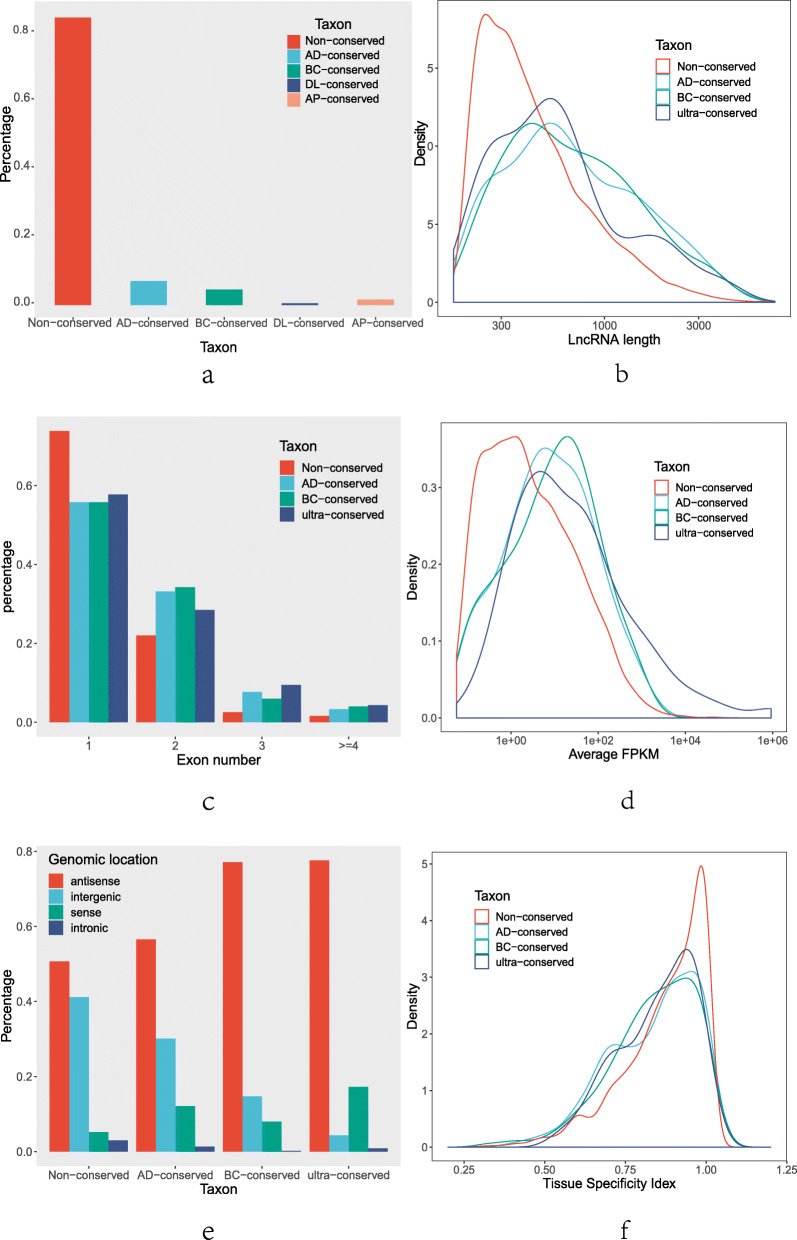
lncRNA classification characteristics. **a** The number of lncRNAs. **b** lncRNA length. **c** The number of lncRNA exons. **d** Average expression levels of lncRNA. **e** lncRNA genomic location. **f** lncRNA tissue specificity index

*A. thaliana* is a representative model plant with a high-quality genome and in which the lncRNAs identified have the advantages of reliability and accuracy. Therefore, understanding the evolutionary history of *A. thaliana* lncRNAs is a key step to further our understanding of the plant lncRNA evolution. We divided *A. thaliana* lncRNAs into five categories based on the degree of conservation: “AD-conserved” lncRNAs conserved in Arabidopsis; “BC-conserved” lncRNAs conserved in Brassicaceae; “DL-conserved” lncRNAs conserved in dicotyledons; “AP-conserved” lncRNAs conserved in angiosperms; “non-conserved” lncRNAs with no conservation. DL-conserved and AP-conserved lncRNAs were combined to avoid statistical errors caused by their small sizes. Collectively these groups are referred to as “Ultra-conserved” lncRNAs. Next, we performed a characteristic analysis across the four categories: Non-conserved, AD-conserved, BC-conserved, and Ultra-conserved. lncRNA number, length, exon number, genomic location, average expression levels (AEL), and tissue specificity index (τ value) were counted separately (Fig. 2).

There were 4677 non-conserved lncRNAs, which accounted for 84.4% of the total lncRNAs (Fig. 2a). There were 389 AD-conserved and 251 BC-conserved lncRNAs, accounting for 7 and 4.5% of the total, respectively. The 28 DL-conserved and 88 AP-conserved lncRNAs were relatively low and accounted for only 2.1% of the total.

Examination of lncRNA sequence length and intron/exon structure revealed that conserved lncRNAs were longer length and contained more exons than did non-conserved lncRNAs. Statistics on the lengths of the lncRNAs across the four categories showed that the density curve of conserved lncRNAs was shifted to the right of that of non-conserved lncRNAs. This result is consistent with the descriptive statistics results that showed the average length of conserved lncRNAs (898 bp) was much longer than that of non-conserved lncRNAs (550 bp) (Fig. 2b). Additionally, all three conserved lncRNA curves showed heavy-tailed distribution. Analysis of the exon numbers across the four lncRNA categories showed that the non-conserved group had a higher proportion of single exon lncRNAs than did the other three groups (Fig. 2c). Conversely, the proportion of multiple exon lncRNAs in conserved lncRNA groups was higher than that in the non-conserved group, and Ultra-conserved lncRNAs accounted for the highest proportion of lncRNAs with exon numbers equal to or greater than three.

lncRNA expression was examined by firstly calculating average expression levels (AEL), where high values reflect high lncRNAs expression levels. The ratio of highly expressed lncRNAs in conserved lncRNAs was much higher than that in non-conserved lncRNAs, and the ratio of highly expressed lncRNAs in the three categories of conserved lncRNAs increased slightly with increased conservation degrees (Fig. 2d). We also calculated the tissue specificity index (τ value), where high values reflect high lncRNAs tissue specificities. We observed a higher proportion of tissue-specific expression in non-conserved lncRNAs than in conserved lncRNAs (Fig. 2f). Using a τ value 0.9 as the threshold, 51% of the non-conserved and 39% of conserved lncRNAs exhibit tissue-specific expression, respectively.

We examined the genomic location of lncRNAs (Fig. 2e) and counted the number of antisense-, intergenic-, sense-, and intronic transcripts in the four categories. The distribution of different transcript types in each lncRNA category were roughly the same, with antisense transcripts accounting for 50.69 to 77.59% of the total, followed by intergenic transcripts (4.31–41.16%), sense transcripts (0.86–2.97%), and intronic transcripts (0–3%). As the degree of conservation increased, the number of antisense transcripts increased and the number of intergenic transcripts decreased. Therefore, we speculated that conserved lncRNAs might be more likely to function as antisense transcripts.

### Functional annotation of conserved lncRNAs based on coding-lncRNA gene co-expression network

The extent of lncRNAs conservation is generally considered to be the key to evaluating their functions. To shed further light on the biological function of conserved lncRNAs, we annotated the functions of 756 conserved lncRNAs from the AD-conserved, BC-conserved, and Ultra-conserved groups based on the theory that the linked gene-pairs in the coding-lncRNA gene co-expression network tended to have more similar annotated functions [14].

To construct the coding-lncRNA *A. thaliana* gene co-expression network, we used the collected RNA-seq datasets to quantify the lncRNA- and coding-gene expression levels. Spearman’s correlation coefficient was used to identify co-expressed gene-pairs from the genes with high expression variation (top 75% percentile). The *P*-value was calculated by Fisher transformation, and *P*-value sets for each gene were corrected using the Bonferroni method. Only gene-pairs with a corrected *P*-value of 0.05 or less were used for subsequent analyses. Finally, we constructed a coding-lncRNA gene co-expression network with 28,730 coding-genes, 384 lncRNA-genes and 38,146,872 edges. Nearly 287,222 edges (0.75%) were linked between coding- and lncRNA-genes, 37,858,201 edges (99.24%) were linked between coding-genes, and another 1449 edges (0.0038%) were connected between pairs of lncRNA-genes.

**Fig. 3:**
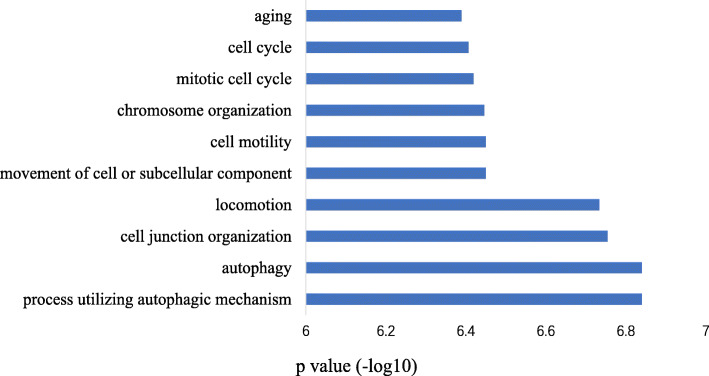
Gene ontology biological process enrichment results for 196 conserved lncRNAs

Each conserved lncRNA was annotated based on its immediate neighbor coding-genes that had previously annotated with at least one gene ontology (GO) Biological Process (BP). Using this approach, we identified 196 of 756 conserved lncRNAs annotated with at least one GO BP term (Additional file 3). We then conducted functional enrichment analysis using a hypergeometric distribution test (*P*-value < 0.05) to explore which functions are performed more often in conserved lncRNAs. The results showed that 39 GO BP terms were significantly enriched in conserved lncRNAs (Additional file 4). The top ten GO BP terms (*P*-value = 6.03e-06) were ‘cell junction organization,’ ‘aging,’ autophagy-related (including ‘process utilizing autophagic mechanism,’ and ‘autophagy’), movement-related (including ‘locomotion,’ ‘movement of cell or subcellular component,’ and ‘cell motility’), and cell-cycle-related (including ‘chromosome organization,’ ‘mitotic cell cycle,’ ‘cell cycle,’ and ‘cytoskeleton organization’) (Fig. 3).

### Functional enrichment analysis of conserved lncRNAs specifically expressed in certain tissues

**Fig. 4:**
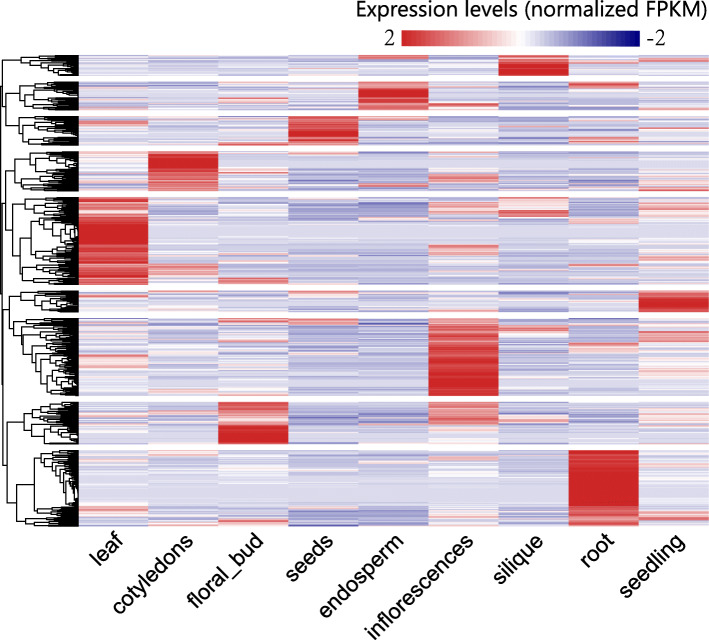
Heatmap of normalized conserved lncRNAs expression levels in nine tissues

Analysis of the expression profiles of the conserved lncRNAs (Fig. 4) revealed that these 756 lncRNAs have obvious tissue specificities. Moreover, their expression patterns fell into nine classes with obvious tissue differences. This phenomenon indicated that the functions of tissue specific lncRNAs were closely related to the growth and development of the corresponding tissues. Therefore, we classified these conserved lncRNAs into nine classes based on the tissue in which they were specifically expressed: leaf-, cotyledons-, floral-bud-, seedling-, seed-, root-, endosperm-, inflorescence-, and silique-expressed lncRNAs. Functional enrichment analysis was then performed for each lncRNA class.

**Fig. 5:**
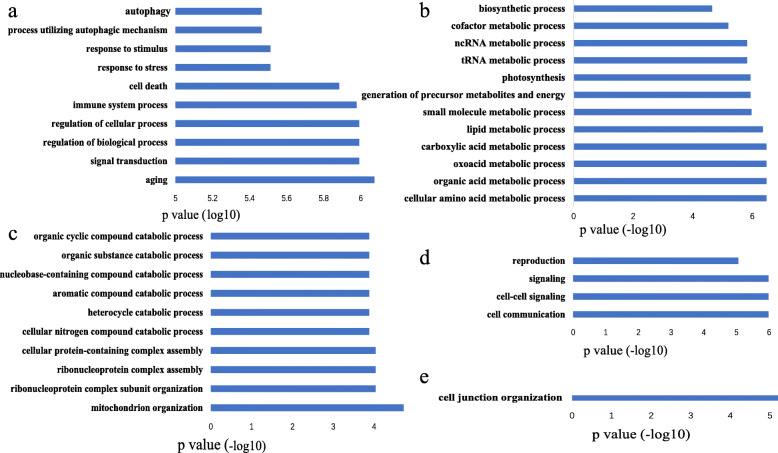
Functional enrichment results (GO BP) of tissue-specific lncRNAs in **a** leaves, **b** cotyledons, **c** seeds, **d** flower-buds, and **e** roots

GO BP enrichment was successful for five of the nine lncRNA classes. We obtained enrichment results of leaf-expressed (Fig. 5a), cotyledons-expressed (Fig. 5b), seed-expressed (Fig. 5c), floral-bud-expressed (Fig. 5d), and root-expressed lncRNAs (Fig. 5e). The enriched functions of lncRNAs specifically expressed in different tissues were closely related to the growth and development of those tissues, which are consistent with our previous speculation.

The top ten enriched GO BP terms for leaf-expressed lncRNAs include ‘aging,’ ‘signal transduction,’ ‘regulation of biological and cellular process,’ ‘immune system process,’ ‘cell death,’ ‘response to stress and stimulus,’ ‘process utilizing autophagic mechanism,’ and ‘autophagy.’ The enriched GO BP terms for cotyledons-expressed lncRNAs include terms closely associated with metabolic process, biosynthetic processes, and photosynthesis. Among them, eight BP terms, including ‘cellular amino acid metabolic process,’ ‘organic acid metabolic process,’ ‘oxoacid metabolic process,’ are relevant to metabolism and the other two are ‘biosynthetic process’ and ‘photosynthesis.’ The enriched GO BP terms for seed-expressed lncRNAs fit into two categories. The first category is protein-complex-assembly-related BP terms, including ‘ribonucleoprotein complex assembly’ and ‘cellular protein-containing complex assembly.’ The second category is metabolic-process-related BP terms, including ‘cellular nitrogen compound catabolic process,’ ‘nucleobase-containing compound catabolic process,’ and ‘mitochondrion organization.’ The top four GO BP terms for floral-bud-expressed lncRNAs are associated with two types of biological processes. One is cell-signaling-related process, including roles like ‘cell communication,’ ‘cell-cell signaling,’ and ‘signaling.’ The other is reproduction processes. For root-expressed lncRNAs, only one GO BP term, ‘cell junction organization,’ was enriched.

## Discussion

As our understanding of lncRNAs develops, it is clear that the study of a single lncRNA can not inform about the nature of all lncRNAs. In pace with the rapid development of high-throughput sequencing technology, the study of lncRNA evolution dynamics has become one of the most important ways to explore lncRNA function. However, most studies on lncRNA evolution have been performed in animals. Very few studies of lncRNA evolution have been performed in plants, in part because plant lncRNAs have high levels of sequence divergence, there is low genome sequence quality, and the systematic collection and characterization of plant lncRNAs is lacking. Here, through large-scale collection and integration of lncRNA data from multiple databases, we obtained a high-confidence plant lncRNA dataset. Using this dataset, we performed the most comprehensive evolutionary analysis of lncRNAs, including the greatest variety of plant species and the most comprehensive lncRNA sequences to date. Our work can not only lay the foundation for further studying of plant lncRNA evolution but also provide clues for studying the specific function of conserved lncRNAs in different plant species.

Unlike protein-coding genes, lncRNA sequences are poorly conserved. Our results confirm the poor conservation of lncRNAs and are consistent with previous reports of lncRNA conservation in various animals and plants [8, 11, 15, 16]. Our results show that the percentage of conserved lncRNA in *A. thaliana* was 15.56%, which is higher than the average proportion of conserved lncRNA in all 25 species of 6.79%. This is probably because we included some species that are closely related to *A. thaliana*. We found that most lncRNAs were traced back to closer ancestors, and they exhibited more conservation than lncRNAs between distant species. This phenomenon is also reflected in the conservation analyses performed by Deng et al. and Washietl et al. [6, 11]. While most lncRNAs do not show conservation across the plant kingdom, it is unclear whether lineage-specific lncRNAs play significant roles in lineage-specific biology. Evolutionary analysis of lncRNAs in tetrapods revealed that 425 (3%) orthologous families originated before 300 Myr. Of the plants we studied, only 90 (1.64%) orthologous families originated before 180 Myr. A study on the evolution of lncRNAs in 17 animal species revealed that more than 70% of lncRNAs appeared before 50 Myr, which is significantly different from the results obtained in this study. This discrepancy may suggest that plant lncRNAs are evolutionarily younger than those of animals.

We specially focused on the differences between *A. thaliana* lncRNAs with conserved and non-conserved sequence. Our results showed that conserved lncRNAs have longer sequences, more exons, and higher expression levels than do non-conserved lncRNAs. Since the biological functions of genes are closely related to their structures, we speculate that conserved lncRNAs may be subjected to greater selection pressure during evolution. This selection pressure leads to the evolution of longer sequences and more exons, which are conducive to their roles in regulating plant growth and development, and that these lncRNAs have then evolved to produce higher expression levels. Additionally, our results show that compared with non-conserved lncRNAs, conserved lncRNAs have lower tissue specificity, and are consistent with the results of previous reports [7, 11]. This finding implies that conserved lncRNAs are more prone to constitutive expression than are non-conserved lncRNAs. This is potentially due to conserved lncRNAs playing a role in regulatory relationships established early in plant evolution. Additionally, the specific expression of lncRNAs in different tissues reflects that they might play important roles in various stages of plant growth and development.

## Conclusions

Through comprehensive integration of large-scale lncRNA data and construction of a phylogenetic tree using lncRNA orthologous families of 25 flowering plants, we conducted the most thorough investigation of plant lncRNA evolutionary history, which is reflected in the origin, conservation, and orthologous relationships of plant lncRNAs. Our focus on the characteristic differences between conserved and non-conserved lncRNAs provides meaningful insights into the unique traits of conserved and non-conserved lncRNAs including their structure, expression, and genomic location. Further functional analysis of the conserved *A. thaliana* lncRNAs revealed tissue specific expression and potential functional roles. Taken together, these results will better our understanding of the evolutionary mechanisms of lncRNAs in plants and provide a platform for further functional studies.

## Methods

### Analysis pipeline

**Fig. 6:**
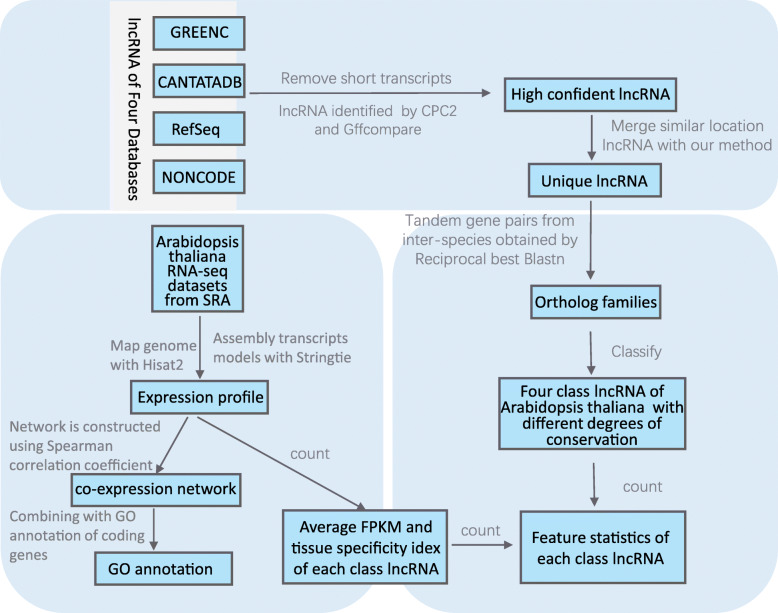
An outline of the data analysis process used in this study

A three-part analysis pipeline was created: 1) lncRNA data collection and integration; 2) lncRNA sequence conservation analysis; and 3) lncRNA function annotation (Fig. 6). The first part of the pipeline included the collection and integration of plant lncRNAs. The second part of the pipeline included construction of the lncRNA ortholog families of 25 flowering plants and comparing the characteristics conserved and non-conserved lncRNAs in *A. thaliana*. The third part of the pipeline was to study the function of conserved and tissue-specific lncRNAs through the construction of a co-expression network.

### Data sources

FASTA sequences for the lncRNAs from 25 plants were downloaded from CANTATAdb2.0 [17], GreeNC v1.12 [18], RefSeq [19], and NONCODEv5 [20] (Additional file 5). Genomic sequences and their corresponding annotations were retrieved from the NCBI genome database [19] (Additional file 1). An RNA-Seq dataset for 90 samples in nine tissues, including leaf, cotyledons, floral bud, seedling, seed, root, endosperm, inflorescence and silique was acquired from the NCBI SRA database [21] (Additional file 6).

### Data integration

All collected lncRNAs were filtered by CPC2 (coding potential) with default parameters and Gffcompare (v0.11.2) (genome location) with an optional parameters “-r” [22, 23]. Then, lncRNAs of each species were mapped to the selected genome version of that species (BLASTN, e-value = 10–5). The clustering method was used to determine the location of each lncRNA on the genome. The fragments of each lncRNA blast results, with a distance of no more than 4000 bp, were clustered, and the effective coverage and weighted identity of each cluster were calculated. We first selected clusters with the effective coverage greater than 70% and the weighted identity greater than 80%, and the clusters with the highest effective coverage under the premise of the highest weighted identity were regarded as the optimal clusters for that particular lncRNA. Multiple optimal clusters were considered paralogous genes. We obtained lncRNA location information based on their optimal clusters so we could merge lncRNAs with overlapping lengths that were greater than 80% of the shorter lncRNA. If multiple lncRNAs were merged, one theoretical lncRNA, containing the union set of these lncRNA exons, was obtained.

### Identification of orthologous families and phylogenetic tree construction

Orthologous gene-pairs were identified based on reciprocal best hits (RBH) with BlastN using a relatively non-stringent E-value threshold of 10–5. The single-linkage clustering method was used to link gene-pairs together to establish orthologous families [7]. Each orthologous family was traced to the nearest branch point of the phylogenetic tree made by TimeTree, the nearest branch point meant that the species at this branch point were the smallest set which could contain all species in the orthologous family [13]. The final step was counting the number of orthologous families per branch point, and adding these numbers onto the phylogenetic tree.

### RNA-seq data analysis pipeline

The SRA-Toolkit was employed to convert SRA format files to FASTQ format files and low quality reads were trimmed using Trimmomatic (Version 0.39) [24]. RNA-Seq reads were aligned to the reference genome using Hisat2 (version 2.1.0) with the parameters of “--min-intronlen 20” and “--max-intronlen 4000.” Stringtie (v1.3.6) was used to calculate lncRNA- and coding-gene expression levels in each sample, which was measured by fragments per kilobase of transcript per million fragments sequenced (FPKM) and scaled by upper-quartile normalization [14, 25].

Using expression profiles of all SRA runs, the average expression level and the tissue specificity index of each lncRNA was calculated using average expression levels (*AEL*) (Eq. 1) and *τ* value (Eq. 2) [26].

1 $$ \mathrm{AEL}=\frac{{\mathrm{AEL}}_{\mathrm{l}}+{\mathrm{AEL}}_{\mathrm{c}}+{\mathrm{AEL}}_{\mathrm{f}}+{\mathrm{AEL}}_{\mathrm{sl}}+{\mathrm{AEL}}_{\mathrm{sd}}+{\mathrm{AEL}}_{\mathrm{r}}+{\mathrm{AEL}}_{\mathrm{e}}+{\mathrm{AEL}}_{\mathrm{i}}+{\mathrm{AEL}}_{\mathrm{sq}}}{9}, $$

The nine parts of the numerator are the expression profile component values of each lncRNA expressed in samples of leaf, cotyledons, floral-bud, seedling, seed, root, endosperm, inflorescence and silique. Each component value was obtained by calculating the mean value of technical repeats and the median value of biological repeats. *AEL* and τ value were only calculated when at least one expression profile component of the expression profile had a value greater than or equal to 0.5.

2 $$ \tau =\frac{\sum_{i=1}^N\left(1-{x}_i\right)}{N-1}, $$

where *N* is the number of tissues and *x*_*i*_ is the expression profile component normalized by the maximal component value. For example, expression profile ‘0 8 0 0 0 2 0 2 0 0 0 0’ is defined to have τ = 0.95. Other definitions, for example, based on entropy or geometric considerations, were pursued but found to be less robust in terms of sensitivity to extreme profile component values.

### Coding-lncRNA gene co-expression network construction

The coding and non-coding genes with high expression variation (top 75% percentile) were retained to construct co-expression network. We employed an in-house Perl script to calculate the Spearman correlation coefficient and its corresponding *P* value between the expression profiles of each gene-pair [14, 25]. Only gene-pairs with an adjusted P value of 0.05 or less were considered to be co-expressed.

### LncRNA functional annotation and enrichment

GO annotation of *A. thaliana* coding gene was downloaded from the Gene Ontology Consortium (only biological process annotations were considered). GO annotation and enrichment of lncRNAs was predicted using goatools (version 0.9.5) [27], which determines the GO annotations of one gene based on the GO annotations of its immediate neighbor genes, and determines enriched GO annotations of study gene list based on the GO annotations of its population gene list (*P*-value < 0.05). Both GO annotation and enrichment are based on hypergeometric distribution (Eq. 3).

3 $$ P=1-{\sum}_{i=0}^{k-1}\frac{\left(\frac{M}{i}\right)\left(\frac{N-M}{n-\mathrm{i}}\right)}{\frac{N}{n}}, $$

For GO annotation, where *N* is the total number of genes in the network, *M* is the total number of genes with at least one GO annotation; *n* is the number of a gene’s immediate neighbors and *k* is the number of neighbor genes with at least one GO annotation. For GO enrichment, where *N* is the total number of annotated genes in the population, *M* is the total number of genes annotated by one certain GO term; *n* is the number of annotated genes in the study and *k* is the number of genes annotated by one certain GO term.

## Supplementary Information

**Additional file 1.** Genomic sequences and their corresponding annotations of 25 flowering plants.

**Additional file 2.** Conserved lncRNAs ratios for each species.

**Additional file 3.** GO annotations of 196 conserved lncRNAs.

**Additional file 4 **Significant enrichment functions (*n* = 39) of conserved lncRNA set (*n* = 196).

**Additional file 5.** Download path of FASTA sequences for the lncRNAs from 25 plants.

**Additional file 6.** The collected transcriptome analysis data.

## Data Availability

FASTA sequences for the lncRNAs are available from CANTATAdb2.0 (https://cantata.amu.edu.pl, https://yeti.amu.edu.pl/CNATATA/), GreeNCv1.12 (https://greenc.sciencedesigners.com/), RefSeq (https://ftp.ncbi.nlm.nih.gov/genomes/refseq/plant/), and NONCODEv5 (http://www.noncode.org/). More detailed download paths for lncRNA sequences of each species can be found in Additional file 5. The processed lncRNA data and data processing scripts are available at https://github.com/changningliu-lab/sang-paper-2021. Genomic sequences and their corresponding annotations were retrieved from the NCBI genome database (https://ftp.ncbi.nlm.nih.gov/genomes/refseq/plant/, Additional file 1). The RNA-Seq dataset was acquired from the NCBI SRA database (https://trace.ncbi.nlm.nih.gov/Traces/sra/, Additional file 6).

## Abbreviations

lncRNA: Long non-coding RNA
RBH: Reciprocal best hits
AEL: Average expression level
FPKM: Fragments per kilobase million
GO: Gene ontology
BP: Biological process
